# Targeting aging pathways with natural compounds: a review of curcumin, epigallocatechin gallate, thymoquinone, and resveratrol

**DOI:** 10.1186/s12979-025-00522-y

**Published:** 2025-07-03

**Authors:** Mohamed Ahmed

**Affiliations:** Department of Pharmacology, Faculty of Veterinary Medicine, Cairo University, Badrachin, 12918 Egypt

**Keywords:** Curcumin, Epigallocatechin gallate, Thymoquinone, And Resveratrol, Aging

## Abstract

Aging is a multifactorial biological process driven by oxidative stress, chronic inflammation, genomic instability, and mitochondrial dysfunction. Recent research underscores the potential of naturally derived compounds to modulate these aging hallmarks. Curcumin, epigallocatechin gallate (EGCG), thymoquinone, and resveratrol exhibit antioxidant, anti-inflammatory, and autophagy-enhancing effects that target core pathways involved in cellular senescence and tissue degeneration. These phytochemicals regulate key molecular players such as sirtuins, AMPK, NF-κB, and mTOR, offering promise in delaying age-associated pathologies and promoting longevity. This review discusses the molecular mechanisms underlying their anti-aging actions and highlights their potential as dietary geroprotective interventions.

## Methodology

This review was conducted to comprehensively evaluate the current evidence on the role of natural bioactive compounds—including curcumin, epigallocatechin gallate (EGCG), thymoquinone, and resveratrol—in promoting healthy aging and longevity. The focus was on their mechanisms of action, particularly regarding antioxidant, anti-inflammatory, autophagy, and mitochondrial-supportive activities.

## Literature search strategy

A systematic search of the scientific literature was performed using electronic databases including PubMed, Scopus, Web of Science, and Google Scholar. The following combinations of keywords and Boolean operators were used:

“curcumin” OR “epigallocatechin gallate” OR “thymoquinone” OR “resveratrol” AND (“longevity” OR “Aging " OR “antioxidant” OR “anti-inflammatory” OR “autophagy” OR “mitochondria” OR “natural compounds” OR “geroprotectors”.

Additional filters were applied to prioritize peer-reviewed articles, systematic reviews, meta-analyses, in vitro and in vivo experimental studies, and clinical trials.

## Inclusion and exclusion criteria

### Inclusion criteria were

Studies evaluating the effects of natural plant-derived bioactive compounds on aging or age-related biomarkers.

Studies exploring mechanisms including oxidative stress reduction, inflammation modulation, autophagy induction, and mitochondrial function improvement.

Articles published in English.

### Exclusion criteria were

Studies focusing solely on synthetic or pharmaceutical compounds.

Articles without available full texts.

Non-peer-reviewed publications such as conference abstracts, editorials, or letters.

Data Extraction and Analysis.

Relevant information extracted from the selected articles included:

The type of bioactive compound studied (curcumin, EGCG, thymoquinone, resveratrol).

Experimental model (cellular, animal).

Effects on antioxidant capacity, anti-inflammatory responses, autophagy pathways, mitochondrial function, and markers of aging and longevity.

Proposed molecular mechanisms and signaling pathways involved (e.g., Nrf2, NF-κB, AMPK, mTOR, SIRT pathways).

A qualitative synthesis approach was used to collate findings, highlighting converging evidence on the roles of these compounds as potential nutritional geroprotectors.

## Introduction

Aging is an inevitable and universal biological phenomenon characterized by the progressive decline of physiological functions and the increased vulnerability to disease and death [1]. At the cellular and molecular levels, aging involves a complex interplay of genetic, epigenetic, metabolic, and environmental factors that contribute to the gradual deterioration of homeostasis [2]. Hallmarks of aging include genomic instability, telomere attrition, mitochondrial dysfunction, epigenetic alterations, deregulated nutrient sensing, cellular senescence, and impaired proteostasis [3, 4]. These alterations collectively compromise tissue regeneration, immune competence, and metabolic efficiency, ultimately leading to the onset of various age-associated diseases [5]. The global demographic landscape is undergoing a dramatic transformation, with a significant rise in the proportion of older adults. According to the World Health Organization (WHO), the global population aged 60 years and older is expected to reach over 2 billion by 2050, nearly doubling from 1 billion in 2020 [6]. This demographic shift poses profound challenges to healthcare systems, social security frameworks, and economic productivity [7]. In light of these challenges, the field of geroscience—aimed at understanding the biological mechanisms of aging and identifying interventions to delay or mitigate its effects—has gained significant momentum [8]. Understanding the underlying processes of aging and exploring potential modulators is crucial for developing effective strategies to promote healthy aging and reduce the societal and economic impact of age-related decline [9].

Increasing attention is being directed toward natural compounds and dietary supplements that exert anti-aging properties, not only to extend lifespan but more importantly to enhance healthspan, the period of life spent in good health [10]. Among these, phytochemical-rich supplements such as curcumin, epigallocatechin gallate (EGCG), thymoquinone, and resveratrol have emerged as promising candidates due to their multifaceted biological properties. These compounds, primarily derived from traditional herbs and foods, have shown efficacy in delaying aging-related degeneration through modulation of cellular signaling pathways, oxidative balance, and inflammation [11, 12]. Curcumin, a polyphenolic compound extracted from *Curcuma longa*, exhibits significant antioxidant, anti-inflammatory, and anti-proliferative activities. Studies suggest that curcumin enhances cellular resilience and modulates pathways linked to longevity including NF-κB and AMPK [13]. EGCG, the major catechin in green tea, has been shown to attenuate cellular senescence and protect against oxidative stress through upregulation of sirtuins and modulation of autophagy-related signaling [14]. Thymoquinone, the bioactive component of *Nigella sativa*, has gained recognition for its immunomodulatory and antioxidant effects, contributing to improved mitochondrial function and reduced systemic inflammation [15]. Resveratrol, a stilbene found in grapes and berries, activates SIRT1 and mimics caloric restriction, enhancing mitochondrial biogenesis and stress resistance [16]. A growing body of evidence from both in vivo and in vitro studies indicates these supplements improve biomarkers of aging, promote telomere stability, and enhance autophagic flux.

## Mechanisms underlying longevity and aging

The aging process is driven by a complex interplay of interconnected cellular and molecular mechanisms, including cellular senescence, epigenetic alterations, loss of proteostasis, and mitochondrial dysfunction [17–19]. Senescence, characterized by irreversible cell cycle arrest, is often triggered by DNA damage, oxidative stress, and telomere shortening [20]. Senescent cells secrete a pro-inflammatory mix of cytokines, chemokines, and proteases known as the senescence-associated secretory phenotype (SASP), which contributes to “inflammaging”—a chronic, low-grade inflammatory state linked to numerous age-related diseases [21]. The progressive accumulation of reactive oxygen species (ROS) disrupts redox homeostasis and accelerates tissue degeneration. Antioxidants—both endogenous and exogenous—counteract this stress and are considered crucial in modulating aging [22, 23]. Inflammation is another critical contributor, with chronic low-grade inflammation (“inflammaging”) being associated with numerous age-related diseases. Elevated pro-inflammatory cytokines such as IL-6 and TNF-α trigger systemic catabolism and impair regenerative mechanisms [24, 25]. Phytochemicals with anti-inflammatory properties can attenuate this process and improve longevity outcomes [26].

Epigenetic modifications, particularly histone acetylation and DNA methylation, also change with age, affecting gene expression and genome stability [27]. The sirtuin family of NAD⁺-dependent deacetylases, especially SIRT1, plays a key role in modulating these epigenetic processes [28]. Sirtuins regulate metabolic homeostasis, DNA repair, and mitochondrial function, and their activation has been associated with lifespan extension in various models [29]. Loss of proteostasis, or the impaired ability of cells to maintain protein quality control, is another hallmark of aging. This is tightly linked to a decline in autophagy, the cellular process responsible for degrading and recycling damaged organelles and misfolded proteins. leading to the accumulation of cellular debris and increased susceptibility to stress [30, 31]. Enhancing autophagy via dietary compounds and exercise has been shown to prolong lifespan in various models [32, 33]. Mitochondrial dysfunction, another hallmark of aging, results in decreased ATP production, increased ROS generation, and activation of cell death pathways [34]. Nutritional supplements that preserve mitochondrial integrity have been shown to support metabolic health and delay senescence [35]. Telomere shortening, a marker of cellular replicative age, is closely linked with genomic instability and aging [36].

## Anti-aging effects of these bioactive compounds

### Resveratrol

Resveratrol, a polyphenol found primarily in red wine, grapes, and several berries, has garnered significant attention due to its purported beneficial effects on lifespan extension and the modulation of age-related diseases. Circadian genes such as Circadian Locomotor Output Cycles Kaput (CLOCK), Brain and Muscle ARNT-Like 1 (BMAL1), Period (PER), and Cryptochrome (CRY) regulate 24-hour cycles of gene expression, hormone release, body temperature, and metabolism. Disruption of these rhythms has been linked to accelerated aging and an increased risk of age-related diseases [37]. It has been well established that resveratrol exerts its anti-aging effects through the activation of sirtuins, modulation of mitochondrial function, and regulation of oxidative stress [38]. Using an in vitro model, Sophie et al. compared the rhythmic gene expression profiles of core clock components, as an indicator of circadian oscillatory function. Resveratrol modulated circadian gene expression in adipose progenitor cells (APCs) across age groups, restoring rhythmicity in components such as CLOCK and CRY, altering patterns in NR1D1 and NR1D2, and disrupting rhythmicity in PER2 and CRY2, highlighting preserved oscillatory function [39]. Resveratrol administration stimulated a notable increase in CD4⁺ T cell populations and enhanced antigen-specific cellular immunity in aged Wistar rats. This intervention also upregulated anti-KLH IgG subclasses, indicating resveratrol’s potential to mitigate immunosenescence and restore adaptive immune competence [40]. Zhu et al. showed that aging resulted in increased ovarian SA-β-gal activity and lipofuscin deposition, but these effects were reversed with resveratrol treatment. Resveratrol increased PCNA expression, enhanced oocyte development, reduced atretic follicles, and regulated gene expression by boosting SIRT1 and NRF2 while downregulating inflammation and ER stress markers in the nothobranchius guentheri model. In SIRT1-deficient HEK293T cells, resveratrol counteracted the heightened inflammation and stress response [41].

### Anti-inflammatory and antioxidant effects

Resveratrol exhibited strong antioxidant activity, as demonstrated by DPPH, ABTS, and ORAC assays, and significantly increased the levels of catalase (CAT), superoxide dismutase (SOD), and glutathione (GSH), while reducing reactive oxygen species (ROS), lactate dehydrogenase (LDH), and malondialdehyde (MDA) in HepG2 cells [42]. The antioxidant activity of resveratrol protects against chondrocyte apoptosis by targeting the COX-2/NF-κB pathway in mice with Temporomandibular joint osteoarthritis (TMJOA) [43]. Moreover, it showed strong anti-inflammatory effects by activating the Nrf2 signaling pathway, thereby attenuating the severity of diabetic neuropathy and protecting neural tissue [44]. Resveratrol, in combination with 5-FU, synergistically intensified oxidative damage in in vitro studies by upregulating ROS production, LPO, and SOD, while concurrently suppressing antioxidant defenses such as catalase and GPx [45]. Downregulation of pro-inflammatory mediators such as TNF-α, IL-8, and MCP-1 by resveratrol occurred following LPS stimulation, without inducing cytotoxicity. This anti-inflammatory effect was supported by transcriptomic and proteomic changes, including decreased NF-κB (p65), LSD1, and APE1/Ref-1 expression, reduced protein acetylation, increased histone methylation, and transcriptional regulation by NRF1 and GABPA [46]. In aged mice, dietary resveratrol exerted anti-inflammatory effects by modulating LPS-induced neuroinflammation, notably decreasing cytokine expression (IL-6, TNF-α, IL-1β, and CXCL10) and regulating key inflammatory mediators such as NF-κB and iNOS, potentially through mTOR pathway inhibition [47]. By inhibiting the TLR2-MyD88-NF-κB signaling pathway, resveratrol was shown to prevent ethanol-induced neuroinflammatory responses [48].

### Sirtuins

In response to environmental stressors such as caloric restriction, sirtuins are activated to help maintain cellular homeostasis and extend lifespan in model organisms like yeast, worms, and mice. RSV counteracted the arsenic-induced downregulation of SIRT1 expression, reestablishing its ability to suppress the senescence-associated protein p16 [49]. The anti-proliferative effects of resveratrol on breast and lung cancer cells involved the induction of cellular senescence, confirmed by increased SA-β-Gal activity and inhibition of colony formation. Its mechanism of action included SIRT1-mediated ER stress, which enhanced p38MAPK and DLC1 expression while reducing NO levels, ultimately leading to genomic instability and mitochondrial dysfunction, hallmark features of senescence [50]. Additionally, resveratrol modulated senescence-associated molecular markers, including p53, p21, and LaminB, in breast and liver cancer cells. Mechanistically, it increased ROS production to upregulate tumor suppressor gene DLC1, which in turn inhibited the DYRK1A–EGFR axis, resulting in DNA damage, evidenced by the upregulation of γH2AX and the downregulation of DNA repair proteins p-BRCA1 and RAD51 [51]. In a study investigating the neuroprotective potential of resveratrol and high-intensity interval training (HIIT), researchers observed significant upregulation of SOD1 and SOD2 protein levels in the frontal lobes of aged rats following the combined intervention of HIIT and resveratrol resveratrol (SOD1: 1.65 ± 0.02; SOD2: 1.7 ± 0.04; *p* < 0.001). Notably, while exercise alone modestly increased SOD1, it significantly reduced SOD2 (0.74 ± 0.08; *p* < 0.05). Combined treatment further enhanced SOD1 (1.74 ± 0.03) and SOD2 (1.43 ± 0.07) expression (*p* < 0.001). Resveratrol also elevated SIRT4 (1.87 ± 0.06; *p* < 0.001) without notably affecting SIRT5, while exercise reduced SIRT4 (0.49 ± 0.01; *p* < 0.001) but increased SIRT5 (1.09 ± 0.009; *p* < 0.05). Combined therapy significantly enhanced SIRT4 (1.63 ± 0.06; *p* < 0.001), with a non-significant increase in SIRT5 [52]. Resveratrol was shown to inhibit premature cellular senescence by modulating RELA (NF-κB subunit) and upregulating SIRT1, indicating its potential role in delaying renal aging and dysfunction [53]. Okudaira et al. demonstrated that resveratrol (RV) completely blocked L1 retrotransposition (L1-RTP) in somatic cells through mechanisms involving PPARα activation, inhibition of MAPK signaling (p38 and CREB phosphorylation), and reduced chromatin recruitment of L1-ORF1 protein. Additionally, RV increased SIRT6 expression and implicated sirtuins SIRT1, SIRT6, and SIRT7, but not SIRT3, in the suppression of L1-RTP activity [54]. The binding affinities and enzymatic activation of SIRT1 and SIRT6 by pinostilbene and resveratrol were analyzed through molecular docking and in vitro assays. The data demonstrated that pinostilbene not only binds similarly to SIRT6 but also activates SIRT1 more efficiently than resveratrol at lower micromolar concentrations (25 µM) [55].

### Mitochondrial health

Resveratrol (20 µM) ‘s contributions to mitochondrial function improvement are evident through its activation of the Sirt1/Sirt3-FoxO pathway, upregulation of protein expression of LC3-II and Mfn2 and increase of Parkin levels in cardiomyocytes, which reestablished mitochondrial fission–fusion dynamics, normalized autophagic activity, reduced intracellular ROS levels, stimulated mitochondrial biogenesis, boosts energy production, and enhances antioxidant defense in H/R cardiomyocytes. These effects were negated by Selisistat (Ex527), a Sirtuin inhibitor [56]. In the context of high-fat diet (HFD)-induced immune dysfunction, resveratrol protected regulatory T cells (Tregs) from apoptosis by reducing oxidative stress, restoring mitochondrial function, and promoting mitochondrial biogenesis [57]. Furthermore, resveratrol exhibits concentration-dependent mitochondrial modulation in HeLa cells, where low doses (≤ 25 µM) decrease ROS levels, suppress LC3-II expression, and enhance mitochondrial respiratory capacity, while high doses (50–100 µM) trigger ROS overproduction, induce autophagy, and impair mitochondrial function, including membrane potential, mtDNA content, and respiration [58]. Lokkin et al.‘s randomized, double-blind, placebo-controlled crossover trial found that 1000 mg/day RSV had no significant effects on VO2max, heart rate, or fatigue scores in adults with mitochondrial myopathy (MM) [59]. However, β-MEND delivery of RSV enhanced maximal mitochondrial respiration and spare respiratory capacity, though baseline respiration remained unchanged [60]. Using diabetic db/db mice and glomerular mesangial cells subjected to high glucose, Zhou et al. reported that RSV was effective in suppressing PDE4D, activating PKA, and inhibiting Drp1-mediated mitochondrial fission at Ser637, an effect disrupted by PDE4D overexpression or PKA [61]. Moreover, RSV facilitated mitochondrial recovery from cryopreservation in oocytes/embryos [62]. Exposure to chronic unpredictable mild stress (CUMS) in C57BL/6J mice led to downregulation of key mitochondrial regulators and neuroprotective pathways, contributing to disrupted neuronal homeostasis and reduced autophagic flux. Resveratrol’s activation of the SIRT1 pathway mitigated these pathological changes by restoring mitochondrial function, enhancing autophagy, and improving GABAergic neurotransmission through the upregulation of SIRT1/PGC-1α/SIRT3 pathway components [63].

### Autophagy

Several studies demonstrate that resveratrol (RSV) modulates autophagy and apoptosis in a context- and dose-dependent manner across cancer and non-cancer models. RSV induces lethal autophagy and apoptosis via the NGFR–AMPK–mTOR axis in tumor cells [64]. Upregulation of LC3 and Beclin1 protein levels following resveratrol treatment indicates enhanced autophagic activity in multiple myeloma cells, highlighting autophagy as a key mechanism. The cytotoxic impact of resveratrol was significantly attenuated by 3-methyladenine (3-MA), an autophagy inhibitor, further confirming the central role of the autophagic pathway in mediating its therapeutic action. Furthermore, resveratrol activated AMPK phosphorylation while downregulating phosphorylated mTOR, p70S6K, and 4EBP1, underscoring the involvement of the AMPK/mTOR axis in mediating autophagy and cytotoxicity [65]. In MM and oral cancer cells, RSV-mediated autophagy is linked to decreased SREBP1 and E-FABP, impairing lipogenesis and selectively targeting malignant cells [66]. In cardiac ischemia-reperfusion (I/R), RSV prevents excessive autophagy through DJ-1-mediated suppression of MEKK1–JNK signaling, reducing LDH release and improving cell viability [67]. Furthermore, RSV activates the AMPK/SIRT1/p-FOXO1 pathway, promotes autophagolysosome formation, and protects mitochondria from damage [68]. Pai et al. demonstrated that resveratrol (RV) impairs homologous recombination (HR)-mediated DNA double-strand break (DSB) repair in breast cancer cells by dual inhibition of AKT signaling and autophagy flux [69]. Using EGFP-LC3 and tf-LC3 reporter systems, RV was shown to block autophagosome–lysosome fusion through lysosomal membrane permeabilization (LMP) [69]. Additionally, RV reversed the LPA-induced suppression of LC3-II accumulation and epithelial-mesenchymal transition (EMT) in SKOV3 (TP53-null) ovarian cancer cells, evident by restored LC3 puncta and E-cadherin re-expression [70].Through inhibition of the Akt/mTOR pathway, resveratrol triggers autophagy, restricting hsBAFF-driven growth and survival in primary murine B lymphocytes and Raji tumor cells; it promotes autophagic flux by enhancing autophagosome formation, upregulating ATG5 and LC3-II, and reducing p62 levels [71] (Table 1).

### Telomere maintenance

Resveratrol exerted anti-senescent effects on endothelial progenitor cells by enhancing telomerase function and Akt phosphorylation, thereby supporting cellular regeneration. The findings suggest that this action is independent of eNOS and estrogen receptors, but partially dependent on the PI3K/Akt signaling cascade, as shown by LY294002 inhibition [72]. In HepG2 hepatocellular carcinoma cells, resveratrol upregulates the expression of human telomerase reverse transcriptase (hTERT) and activates the SIRT1/Nrf2 signaling pathway in a dose-dependent manner [73]. Treatment with 5 nmol/L resveratrol for 48 h significantly reversed senescence markers in MSCs at later passages, as demonstrated by reduced SA-β-Gal staining and increased proliferation. The effect was associated with upregulation of hTERT mRNA and suppression of p-mTOR (Ser2448), implying a dual role of RSV in enhancing telomerase activity and inhibiting pro-senescent mTOR signaling [74]. Female rats receiving RSV showed significantly longer hepatic telomeres after 9 and 21 months of treatment—8.29 ± 0.17 kb and 8.92 ± 0.14 kb—compared to untreated females at 7.71 ± 0.14 kb and 8.06 ± 0.42 kb (*p* < 0.05). MEL had no such effect in females, and in 2-year-old males, it reduced liver telomere length from 9.22 ± 0.51 kb to 8.32 ± 0.25 kb (*p* < 0.05); neither treatment altered kidney telomere lengths [75].

**Table 1:** A summary of resveratrol’s effects on aging-related pathways

Model	Treatment/Duration	Main findings	Reference
in young and older human adipose-derived progenitor cells	Resveratrol (100 µM, 48 h)	Modulated circadian gene expression; restored CLOCK and CRY rhythmicity ↑; disrupted PER2 and CRY2 rhythmicity ↓	[39]
short-lived fish	(200 µg/g)	↑ SIRT1, ↑ NRF2; ↓ NF-κB, ↓ IL-1β, ↓ TNF-α, ↓ IL-8, ↓ GRP78, ↓ CHOP.	[41]
HepG2 cells	Resveratrol	Catalase (CAT) ↑, SOD ↑, GSH ↑; ROS ↓, LDH ↓, MDA ↓	[42]
Mice with TMJOA	Resveratrol.	↓ COX-2/NF-κB pathway	[43]
Diabetic neuropathy model	Resveratrol.	Nrf2 pathway activation ↑; neural inflammation ↓	[44]
In vitro studies	Resveratrol + 5-FU	ROS ↑, LPO ↑, SOD ↑; Catalase ↓, GPx ↓	[45]
LPS-stimulated H3K9 cells	Resveratrol.	TNF-α ↓, IL-8 ↓, MCP-1 ↓; NF-κB (p65), LSD1, APE1/Ref-1 ↓; NRF1 and GABPA transcription regulation ↑	[46]
Aged mice with neuroinflammation	Resveratrol.	IL-6 ↓, TNF-α ↓, IL-1β ↓, CXCL10 ↓; NF-κB and iNOS downregulation; mTOR pathway inhibition	[47]
Ethanol-induced neuroinflammation	Resveratrol.	Inhibited TLR2-MyD88-NF-κB signaling pathway	[48]
Sprague-Dawley rats (arsenic model)	Resveratrol. (20 mg/kg for 36 weeks)	SIRT1 ↑, p16 ↓	[49]
Breast and lung cancer cells	R (0, 10, 20 µM for 10 days)	SA-β-Gal activity ↑; ER stress ↑ (via SIRT1); p38MAPK and DLC1 ↑, NO ↓	[50]
Breast and liver cancer cells	Resveratrol.	p53 ↑, p21 ↑, LaminB ↑, ROS ↑, DLC1 ↑; DYRK1A–EGFR axis ↓; γH2AX ↑, p-BRCA1 and RAD51 ↓	[51]
Aged rats + HIIT	(10 mg/kg/day, 6 weeks)	SOD1 ↑, SOD2 ↑, SIRT4 ↑; Exercise alone reduced SIRT4 ↓	[52]
H2O2-induced senescence of BMMSCs	Resveratrol (1, 5, 10 µM, 24 h)	RELA modulation ↓, SIRT1 ↑	[53]
Somatic cells (L1-RTP study)	Resveratrol.	PPARα activation ↑, MAPK inhibition ↓, SIRT6 ↑; L1-ORF1 chromatin binding ↓	[54]
In vitro sirtuin activation	Resveratrol (≤ 25 µM)	SIRT1 and SIRT6 activation ↑ (pinostilbene stronger for SIRT1)	[55]
Cardiomyocytes (H/R injury)	Resveratrol (20 µM, 12 h)	LC3-II ↑, Mfn2 ↑, Parkin ↑, mitochondrial fission–fusion restored, ROS ↓	[56]
HFD-induced immune dysfunction	Resveratrol.	Oxidative stress ↓, mitochondrial biogenesis ↑, Treg survival ↑	[57]
HeLa cells	R (≤ 25 µM, 24 h; high doses 50–100 µM)	Low doses: ROS ↓, LC3-II ↓; High doses: ROS ↑, autophagy ↑, mitochondrial damage ↑	[58]
Adults with mitochondrial myopathy	Resveratrol (1000 mg/day)	No significantn significant differences in HR, or fatigue changes	[59]
β-MEND + Ra in vitro	Resveratrol.(10 µM)	Maximal mitochondrial respiration ↑, spare respiratory capacity ↑	[60]
Diabetic nephropathy model	Resveratrol	Drp1-mediated fission ↓ via PDE4D, PKA, Drp1 regulation	[61]
Oocytes/embryos (cryopreservation)	Resveratrol	↑ mitochondrial biogenesis and degradation	[62]
CUMS-induced molecular and behavioral alterations in mice	Resveratrol (30 mg/kg, 3 weeks)	PGC1α ↑, SIRT3 ↑, GABA signaling regulation ↑	[63]
Tumor models	Resveratrol (40–140 µM, 48 h)	Lethal autophagy and apoptosis ↑ via NGFR–AMPK–mTOR axis	[64]
MM model	Resveratrol (100 µM, 48 h)	LC3 and Beclin1 ↑, autophagy ↑; 3-MA reversed effects	[65]
Oral cancer cells	Resveratrol (50 µM, 72 h)	SREBP1 ↓, E-FABP ↓, lipogenesis ↓	[66]
Cardiac I/R injury	Resveratrol (20 µM, 24 h)	DJ-1-mediated MEKK1–JNK suppression ↓, LDH release ↓, cell viability ↑	[67]
Endothelial cells	Resveratrol (0.1–1 µM, 30 min)	AMPK/SIRT1/p-FOXO1 activation ↑, mitochondrial protection ↑	[68]
Breast cancer cells	Resveratrol (50 mg/kg, 48 h)	HR-mediated DSB repair ↓ via AKT and autophagy flux inhibition	[69]
SKOV3 ovarian cancer cells	Resveratrol.(100 µM, 72 h)	LC3-II accumulation ↑, EMT reversal ↑	[70]
B lymphocytes and Raji tumor cells	Resveratrol	hsBAFF-driven growth ↓ via Akt/mTOR inhibition, autophagy flux ↑ (ATG5 ↑, LC3-II ↑, p62 ↓)	[71]
Endothelial progenitor cells	Resveratrol (RSV)	↑ Telomerase activity and ↑ Akt phosphorylation; effect independent of eNOS and ERs, but partially dependent on PI3K/Akt (blocked by LY294002)	[72]
HepG2 hepatocellular carcinoma cells	Resveratrol (RSV)	↑ hTERT expression and ↑ SIRT1/Nrf2 signaling in a dose-dependent manner	[73]
MSCs (late passage)	RSV (5 nmol/L, 48 h)	↓ SA-β-Gal staining, ↑ proliferation, ↑ hTERT mRNA, and ↓ p-mTOR (Ser2448); suggests dual effect on telomerase activation and mTOR inhibition	[74]
Female and male rats (aged)	RSV and Melatonin (MEL)	In females: RSV ↑ liver telomere length; MEL had no effect. In males: MEL ↓ liver telomere length. Neither treatment altered kidney telomere length	[75]

## Curcumin

Curcumin, a polyphenolic compound derived from the turmeric root, has garnered significant attention for its potential therapeutic properties, particularly in the context of aging. Curcumin has demonstrated anti-inflammatory, antioxidant, and anti-cancer properties, making it a promising candidate for interventions in aging and age-related diseases [76]. Curcumin promotes longevity in C. elegans by reducing oxidative stress, enhancing mitochondrial DNA replication, and modulating MAPK signaling pathways through the upregulation of oxidative stress-related genes (sod-1, sod-2, sod-3, gst-4) and the downregulation of MAPK pathway-related genes (sek-1, pmk-1, nsy-1) [77]. The anti-aging potential of curcumin (200 mg/kg b.w., oral) was demonstrated in a D-galactose-induced rat model, where it enhanced antioxidant defenses, improved mitochondrial function, and upregulated aging-related gene expression such as SIRT-1, autophagy-associated genes Beclin-1 and ULK-1, and the neuroprotective marker NSE, while simultaneously downregulating pro-inflammatory cytokines IL-6 and TNF-α in brain tissue [78].

### Antioxidant and anti inflammatory

Curcumin was shown to inhibit the expression of pro-inflammatory cytokines such as IL-1β, IL-6, and TNF-α, and suppress the activation of NF-κB and MAPK signaling pathways in both in vitro and in vivo models of inflammation and arthritis [79, 80].Curcumin modulated macrophage polarization through AMPK signaling, thereby mitigating inflammation associated with myocardial infarction (MI). Following curcumin administration, MI mice exhibited reduced myocardial expression of proinflammatory markers and enhanced IL-10 production compared to untreated MI controls [81]. Fe-Cur NPs, delivered intravenously in ALI mice, effectively lowered TNF-α, IL-1β, and IL-6 levels, restricted Ca²⁺ mobilization, blocked NLRP3 inflammasome activity, and downregulated NF-κB pathway activation [82]. Curcumin’s anti-inflammatory effects were achieved through suppression of MAPK signaling (ERK, p38, Akt) and inhibition of NF-κB activation following LTA stimulation in microglia [83, 84]. It also upregulated HO-1 and Nrf2 expression, with HO-1 playing a crucial role, as its inhibition counteracted curcumin’s benefits [84]. Several studies focused on nanocarriers (e.g., chitosan, polysaccharides, and amphiphilic polymers) that improved curcumin’s bioavailability, antioxidant capacity (e.g., increased SOD, CAT, GPx), and stability while maintaining low toxicity [85–88]. A combination therapy using curcumin and vitamin C synergistically protected against methotrexate-induced liver injury via boosted antioxidant action [89]. Overall, these findings emphasize that curcumin acts on multiple molecular targets, making it an effective therapeutic candidate for inflammation- and oxidation-related diseases, especially when formulated with advanced delivery systems.

### Telomere maintenance

Telomeres, the protective caps at the ends of chromosomes, shorten with each cell division and are associated with cellular aging. Telomere shortening has been linked to several age-related diseases, including cardiovascular disease, diabetes, and neurodegeneration [90]. Curcumin has been reported to inhibit telomerase action in tumor cells and induces telomere shortening [91–93].

### Mitochondrial function

Curcumin’s impact on mitochondrial function has been extensively studied. Research indicates that curcumin is capable of improving mitochondrial biogenesis and function via activating key signaling pathways, such as the SIRT1 pathway, which plays a crucial role in mitochondrial health and longevity [94]. In one in vivo study, Curcumin protects against sepsis-induced cardiac dysfunction by lowering excessive mitochondrial fission and enhancing mitochondrial biogenesis through the upregulation of PGC-1α, TFAM, and NRF2. This process restores mitochondrial morphology and function in cardiac cells of LPS-induced mouse models. It also enhanced mitochondrial dynamics by regulating the mitochondrial translocation of DRP1 via SIRT1 activation in septic cardiomyocytes [95]. Moselhy et al. investigated the protective effects of curcumin nanoparticles (CNPs) against γ radiation-induced mitochondrial dysfunction and cellular senescence in rat brain tissue. Oral CNP administration (10 mg/kg/day, three times per week for eight weeks) markedly enhanced mitochondrial performance by improving the activities of complex I, complex II, and ATP production. In irradiated rats, CNPs significantly decreased brain β-galactosidase activity and the expression of senescence markers p53, p21, and p16 (*P* < 0.05) while significantly upregulating AMPK mRNA expression [96]. Zhou et al. demonstrated that curcumin protects against thapsigargin-induced endoplasmic reticulum stress (ERS), oxidative stress, and mitochondrial dysfunction by downregulating GRP78, pSer981-PERK, and pSer51-eIF2α protein levels, in a mechanism dependent on mitofusin-2 (Mfn2) regulation [97]. Curcumin treatment in 5/6 nephrectomized rats improved mitochondrial respiration and prevented membrane depolarization, enhancing oxidative phosphorylation capacity. Additionally, Curcumin upregulated PPARα and CPT1 expression, partially restoring fatty acid β-oxidation, despite persistent reductions in PGC1α, VDAC, and ATP5a levels [98].

### Autophagy

Autophagy, the process by which cells degrade and recycle damaged organelles and proteins, is crucial for maintaining cellular homeostasis and delaying the aging process. Dysregulated autophagy has been linked to several age-related diseases, including neurodegenerative disorders [99]. Curcumin has been reported to modulate autophagy across various models by influencing key markers and pathways. Zhang et al. reported that curcumin induced autophagy in thyroid cancer cells, increasing LC3-II, Beclin-1, AVO formation, and p62 degradation, with autophagy inhibition via 3-MA partially rescuing the cells [100, 101]. Ye and colleagues developed a rat model of knee osteoarthritis (KOA) and treated the animals with curcumin for five weeks to investigate its effects. They assessed autophagy flux, oxidative stress by detecting ROS levels, and SIRT3-SOD2 expression in quadriceps femoris tissue. Curcumin administration induced upregulation of SIRT3, reduction of SOD2 acetylation, and suppression of excessive autophagy and ROS levels [102]. In gastric cancer (GC) cells, Zheng et al. showed that curcumin increased ATG5, ATG7, Beclin1, LC3B, and modulated ferroptosis through iron, MDA, ACSL4, and reduced SLC7A11 and GPX4, while suppressing the PI3K/AKT/mTOR pathway [103]. Hanafy et al. further observed that curcumin-niacin nanoparticles enhanced mTOR and p62 mRNA expression in HepG2 cells, confirming autophagy activation via nanodelivery systems [104]. In renal epithelial cells exposed to D-galactose-induced stress, curcumin significantly reduced senescence-associated β-galactosidase, PCNA, and markers of oxidative stress such as ROS and 8-OHdG. This response was observed as a 20% decrease in β-gal in LLC-PK1 cells, 60% decline in PCNA in HK-2 cells, and a general 20–25% reduction in oxidative indicators [105]. Enhancement of autophagosome and autolysosome formation by curcumin promotes autophagy in D-galactose-induced senescent cardiomyocytes, accompanied by a dose-dependent increase in the LC3 II/I ratio and a reduction in p62 protein levels through activation of the SIRT1/AMPK/mTOR pathway [106].

### Nutrient-Sensing pathways

Nutrient-sensing pathways, including those regulated by insulin/IGF-1, mTOR, and sirtuins, play a crucial role in the aging process. Curcumin has been shown to modulate several of these pathways, contributing to its anti-aging effects. Curcumin downregulated IGF-1R and inactivated the PI3K/Akt pathway in CRPC cells and tumor tissues, leading to growth inhibition and apoptosis induction [107]. The combination of orlistat and curcumin effectively modulated nutrient-sensing pathways by influencing SKN-1/NRF-2 activity in Caenorhabditis elegans [108]. Curcumin treatment in atherosclerotic rats showed a marked decrease in aortic SA-β-gal activity and significantly reduced the serum concentration of the pro-inflammatory marker MCP-1 [109]. Branchedchain amino acid transaminase 1 (BCAT1), an aminotransferase enzyme, plays a significant role in cancer cell progression. Its interference with cancer cell proliferation has been linked to the regulation of mTOR-mediated mitochondrial biogenesis and function. Curcumin downregulated BCAT1 expression across several myeloid leukemia cell lines, including both parental and cytarabine-resistant variants, while its metabolite tetrahydrocurcumin and cytarabine itself had no such effect. A bidirectional interaction between BCAT1 and mTOR signaling pathways was observed specifically in cytarabine-resistant HL60 cells [110]. Curcumin induces p16^INK4A-dependent, DNA damage-independent senescence in proliferating breast stromal fibroblasts without triggering an inflammatory secretory phenotype, while downregulating JAK2/STAT3 signaling and suppressing pro-tumorigenic markers such as α-SMA, IL-6, SDF-1, MMP-2, MMP-9, and TGF-β [111]. Young rats subjected to constant light showed a phenotype resembling natural immunosenescence, characterized by suppressed antioxidant defenses and heightened oxidative and inflammatory responses. Both melatonin and turmeric counteracted these effects by upregulating Nrf2/DJ-1 and downregulating p53/Bax signaling [112]. Besides, curcumin promotes apoptosis by inhibiting key signaling pathways like PI3K/AKT and JAK/STAT, leading to downregulation of anti-apoptotic proteins such as Bcl-2 and upregulation of pro-apoptotic markers like Bax and caspase-8. This effect is mediated through mechanisms including PTEN activation, EGFR inhibition, and sensitization of cells to death receptor pathways and endoplasmic reticulum stress [113] (Table 2).

**Table 2:** A summary of curcumin’s effects against aging-related pathways

Model	Treatment/Duration	Main Findings (with ↑ ↓ markers)	References
C. elegans	Curcumin	↑ Oxidative stress resistance (↑ sod-1, sod-2, sod-3, gst-4); ↓ MAPK signaling (↓ sek-1, pmk-1, nsy-1)	[77]
D-galactose-induced rats	Curcumin (200 mg/kg b.w., oral)	↑ Antioxidant defense, mitochondrial function, SIRT-1, Beclin-1, ULK-1, NSE; ↓ IL-6, TNF-α	[78]
In vitro and in vivo inflammation/arthritis models	Curcumin	↓ IL-1β, IL-6, TNF-α; ↓ NF-κB and MAPK activation	[79, 80]
Myocardial infarction mice	(100 mg/kg/day, i.p., 6 weeks)	↓ Pro-inflammatory markers; ↑ IL-10 via AMPK signaling modulation	[81]
Acute lung injury mice (Fe-Cur NPs)	25 µg/mL, i.v.	↓ TNF-α, IL-1β, IL-6; ↓ Ca²⁺ mobilization; ↓ NLRP3 inflammasome; ↓ NF-κB activation	[82]
LTA-stimulated microglia	25 mg/kg for 6 days	↓ ERK, p38, Akt (MAPK pathway); ↑ HO-1, Nrf2 (HO-1 critical for effect)	[83, 84]
Nanocarrier studies	Various (e.g., chitosan, polymers)	↑ SOD, CAT, GPx; ↑ Bioavailability and stability of cisplatin	[85–89]
Methotrexate-induced liver injury (VC + cisplatin)	Vitamin C + curcmin100 mg/kg + cisplatin 10 mg/kg for 10 days	↑ Antioxidant action; Synergistic protection	[90]
(tumor cells)	curcmin	↓ Telomerase activity; ↑ Telomere shortening	[91–93]
Sepsis-induced cardiac dysfunction (mice)	Curcmin (80 mg/kg for 4 weeks)	↓ Mitochondrial fission; ↑ PGC-1α, TFAM, NRF2; ↑ SIRT1-mediated mitochondrial biogenesis	[94]
γ-radiation-induced brain aging (rats, CNPs)	Curcmin (10 mg/kg/day, 3x/week, 8 weeks)	↑ Complex I, II activities, ATP; ↓ β-galactosidase, p53, p21, p16; ↑ AMPK expression	[95]
ER stress model (cell culture)	(curcmin) 2.5 µmol/L for 4 h	↓ GRP78, pSer981-PERK, pSer51-eIF2α via Mfn2 regulation	[96]
5/6 nephrectomy rats	120 mg/kg for 4 weeks	↑ PPARα, CPT1; Improved mitochondrial respiration; Partial fatty acid β-oxidation recovery	[98]
Thyroid cancer cells		↑ Autophagy (↑ LC3-II, Beclin-1, ↓ p62)	[100, 101]
KOA-induced skeletal muscle atrophy	Curcumin (150 mg/kg/day for 5 weeks)	↑ SIRT3, ↓ SOD2 acetylation; Balanced autophagy and ROS	[102]
Gastric cancer cells	curcmin	↑ ATG5, ATG7, Beclin1, LC3B; Modulated ferroptosis; ↓ PI3K/AKT/mTOR pathway	[103]
HepG2 cells (cisplatin-niacin nanoparticles)	curcmin	↑ mTOR, p62 mRNA; Enhanced autophagy activation	[104]
Renal epithelial cells (D-galactose stress)	Curcmin (5 µM for 120 h)	↓ β-galactosidase, PCNA, ROS, 8-OHdG	[105]
Senescent cardiomyocytes (D-galactose-induced)	Curcmin (1–10 µM for 72 h)	↑ LC3-II/I ratio; ↓ p62 via SIRT1/AMPK/mTOR activation	[106]
CRPC cells and tumors	Curcmin (100 mg/kg, 3x/week, 4 weeks)	↓ IGF-1R; ↓ PI3K/Akt; Induced apoptosis	[107]
C. elegans	(orlistat + curcumin)	Modulated SKN-1/NRF-2 activity; Anti-aging effect	[108]
Atherosclerotic rats	curcmin	↓ SA-β-gal activity; ↓ MCP-1	[109]
AML cell models	Curcmin (0–50 µM for 2–48 h)	↓ BCAT1; Disruption of BCAT1–mTOR axis; Apoptosis induction	[110]
Breast stromal fibroblasts	Curcumin (10 µM for 24 h)	↑ p16^INK4A; ↓ JAK2/STAT3; ↓ α-SMA, IL-6, SDF-1, MMP-2/9, TGF-β	[111]
In vivo and in vitro studies	curcmin	Apoptosis promotion (↓ PI3K/AKT, ↓ JAK/STAT; ↑ Bax, caspase-8; ↓ Bcl-2)	[113]

## Epigallocatechin gallate

Epigallocatechin gallate (EGCG), the most abundant catechin in green tea, has demonstrated a wide array of beneficial biological effects, including antioxidant, anti-inflammatory, anti-cancer, and anti-aging properties [114]. Due to its ability to modulate several molecular pathways involved in cellular aging, EGCG has emerged as a promising candidate for anti-aging therapy. C. sinensis extracts and their active compound EGCG have been shown to enhance mitochondrial electron transport and oxidative phosphorylation efficiency, lower ROS production, and eliminate senescent fibroblasts, thereby contributing to skin aging reversal and barrier repair [115]. Sharma et al. performed an in vivo study to investigate the progression of cellular senescence across multiple mouse organs at four distinct life stages and examined how EGCG influences markers of senescence, inflammation, immune decline, and gut microbial dysregulation. Prolonged EGCG supplementation markedly reduced indicators of DNA damage, cell cycle arrest, and regulators of the senescence-associated secretory phenotype, while modulating AMPK/AKT signaling and promoting SIRT3/5 expression and autophagy. It also lowered systemic inflamm-aging markers, enhanced early T cell activation, and preserved gut microbial diversity by decreasing pathogenic bacterial populations [116]. The anti-aging effects of EGCG were observed in a post-ovulatory aging model using porcine Metaphase II (MII) oocytes cultured in vitro for 48 h with varying concentrations of EGCG (0–100 µM). EGCG significantly mitigated aging-induced oxidative stress, prevented glutathione (GSH) depletion, and reduced apoptosis and autophagy. Additionally, EGCG supplementation preserved mitochondrial function by decreasing mitochondrial DNA copy number while increasing the number of active mitochondria and adenosine triphosphate (ATP) levels, thereby supporting oocyte viability and developmental competence during aging [117].

### Antioxidant and Anti-inflammatory effects

It has been reported the antioxidant and anti-inflammatory effects of epigallocatechin-3-gallate (EGCG) through modulation of multiple molecular pathways across various models. EGCG exerts anti-inflammatory effects in LPS-stimulated BV-2 microglia by inhibiting NO and IL-6 secretion and promoting TNF-α expression. EGCG also suppresses the mRNA expression of mTOR, NF-κB2, STAT1, Akt3, CCL5, and SMAD3, while enhancing the expression of Ins2, Pld2, A20/TNFAIP3, and GAB1 [118]. By inhibiting the ERK1/2, p38 MAPK, and NF-κB signaling pathways, EGCG alleviated oxidative stress, prevented antioxidant depletion, lowered IL-8 levels, and reduced apoptosis in cardiomyocytes induced by cigarette smoke [119]. In an in vitro study, EGCG markedly suppresses the expression of inflammatory cytokines such as TNF-α, IL-6, IL-8, and IL-1β [120]. Keratinocytes exposed to interface dermatitis (ID)-like stimuli showed high levels of CXCL10 and MxA, mirroring their strong presence in lichen planus lesions. EGCG treatment not only decreases these markers but also curbs pro-inflammatory signaling and protects against stimulus-induced cytotoxicity [121]. Recent research has shown that Nanoparticle-based EGCG formulations enhanced its therapeutic efficacy and bioavailability. For instance, liposomal EGCG effectively reduced vascular inflammation and thrombosis markers such as VCAM-1 and E-selectin in tumor endothelial models, while EGCG-loaded liposomes showed anti-inflammatory activity in microglia and supported neuroprotection [120]. EAC nanoparticles, formed by chelating Cu²⁺ with epigallocatechin gallate, confer the collagen scaffold with enhanced bioactivity—such as reactive oxygen species (ROS) scavenging, and inflammation mitigation in an in vivo study. These nanoparticles also allow for controlled Cu²⁺ release under oxidative stress and preserve the scaffold’s porous architecture [122]. Comparative studies further showed enhanced antioxidant and anti-inflammatory effects when EGCG was encapsulated with phosphatidylcholine or combined with curcumin in nanodelivery systems [123, 124]. In cancer and chemotherapy models, EGCG inhibited ROS-related DNA damage and NF-κB/STAT3 signaling, enhancing anticancer effects [125]. Furthermore, EGCG nanoparticles demonstrated improved antioxidant stability and Nrf2-mediated hepatic REDOX regulation [126].

### Mitochondrial function

EGCG was found to restore mitochondrial homeostasis in cardiac hypertrophy by modulating epigenetic and transcriptional pathways, particularly through the inhibition of HDAC1. This leads to the upregulation of key mitochondrial regulators such as TFAM and FUNDC1, restoration of mitochondrial DNA content, improved mitophagy, and enhanced cardiac function. These protective effects are linked to increased histone acetylation at mitochondrial gene promoters and strengthened NRF1–PGC-1α interactions. HDAC1 overexpression blocked the resveratrol-induced upregulation of NRF1, TFAM, and FUNDC1 in neonatal cardiomyocytes [127]. Modulation of VGCC-mediated calcium entry by EGCG alleviates SAH-induced mitochondrial damage, preserves mitochondrial membrane potential, and balances dynamics-related proteins such as Drp1, OPA1, Mfn1/2, and Fis1. Additionally, EGCG promotes autophagic clearance of damaged mitochondria, prevents neuronal apoptosis, and improves functional recovery [128]. EGCG (9 mg/kg) downregulated ERK activity and cleaved-caspase 3 expression while upregulating ATPase expression in the CA/CPR model. It also decreased mPTP opening and ROS levels, and increased ATP content, indicating restored mitochondrial function in the CA/CPR model [129]. In the AβPP/PS-1 transgenic mouse model of Alzheimer’s disease, EGCG treatment significantly normalized mitochondrial respiratory function, membrane potential, ROS balance, and ATP content by 50–85% in the hippocampus, cortex, and striatum [130]. EGCG also suppressed both mRNA and protein expression of mitophagy-related factors and inhibited osteoclast differentiation by modulating mitophagy via the AKT and p38MAPK signaling pathways [131]. Using in vivo animal models, kim et al. reported that EGCG enhanced mitochondrial membrane potential and downregulated Drp1 expression. It also shifted cellular metabolism toward oxidative phosphorylation by modulating the activities of malate dehydrogenase (MDH) and hexokinase (HK), compared to DOX-treated controls [132]. Kim et al. found that EGCG counteracted mitochondrial hyperfusion by preserving ERK1/2 and DRP1-mediated DRP1 mitochondrial fission without involving JNK activity. It also suppressed phosphorylation of NF-κB at Ser536, contributing to protection against SE-induced neuronal loss in the CA1 region [133]. Alleviation of heat-stress-induced fat accumulation in porcine subcutaneous preadipocytes by EGCG involves targeting HSP70 and activating the AMPK-SIRT1-PGC-1α signaling pathway [134].

### EGCG and autophagy

EGCG has been shown to activate autophagy and autophagic activator in type 2 diabetic rats as, shown by upregulated expression of LC3, Beclin1, and p-AMPK and decreased expression of the p-mTOR [135]. In cellular models, EGCG has been demonstrated to increase the number of autophagosomes, thus improving autophagic flux and the clearance of damaged cellular components [136]. In experimental stroke models, EGCG inhibited autophagy through modulation of the AKT/AMPK/mTOR phosphorylation cascade. The reversal of these effects by GSK690693 and rapamycin confirmed EGCG’s involvement in regulating this pathway [137]. EGCG (25 mg/kg, i.p. for 42 days) inhibited extracellular HMGB1 expression and suppressed inflammasome activation in rcccDNA mice. This effect was driven by the activation of autophagy, promoting the degradation of cytoplasmic HMGB1 in hepatocytes [138]. Lee et al. showed that the EGCG derivative E10 significantly promoted autophagic flux in HAECs via AMPK phosphorylation, outperforming native EGCG. This enhanced autophagy conferred protection against lipotoxic damage, senescence, and oxidative stress-induced apoptosis [139]. EGCG reversed TGF-β1-induced suppression of autophagy and significantly reduced the expression of the fibrosis marker α-SMA, supporting its role in antifibrotic signaling [140]. EGCG enhanced autophagy in lung tissues of LPS-induced pneumonia models by downregulating mTOR expression and upregulating LC3 and BECN1 levels [141]. EGCG facilitated the degradation of FTO via the ubiquitin–proteasome system in NR3C1-enhanced β cells, reducing oxidative stress and preventing excessive autophagy. However, overexpression of FTO negated the protective effects of EGCG on β cells [142]. EGCG also increased autophagy-related proteins including Atg5, Atg7, LC3 II/I, and the Atg5–Atg12 complex and downregulated the expression of proteins linked to apoptosis in HUVECs. This was accompanied by suppression of the PI3K-AKT-mTOR pathway [143].

### Telomere maintenance

EGCG was shown to increase senescence due to telomere shortening as well as induce genotoxicity through telomere-independent mechanisms [144]. Under oxidative conditions induced by H₂O₂, EGCG significantly blocked telomere erosion, loss of the shelterin protein TRF2, and the upregulation of senescence markers p53 and p21 [145]. EGCG treatment led to telomere shortening and suppression of telomerase activity in Caco-2 cells, while in fibroblasts, it promoted telomere length maintenance and increased methylation at six CpG sites within the hTERT promoter region, potentially influencing telomerase gene expression epigenetically [146] (Table 3).

**Table 3:** Summarizes the potential effects of Epigallocatechin gallate against aging-related pathways

Model	Treatment/Duration	Main findings	References
LPS-stimulated BV-2 microglia	EGCG (150 µM for 24 h)	↓ NO, IL-6; ↑ TNF-α; ↓ mTOR, NF-κB2, STAT1, Akt3, CCL5, SMAD3 mRNA; ↑ Ins2, Pld2, A20/TNFAIP3, GAB1 expression	[118]
cigarette smoke-induced inflammation in human cardiomyocytes	EGCG treatment	↓ oxidative stress, IL-8, apoptosis; ↑ antioxidant defense; inhibition of ERK1/2, p38 MAPK, NF-κB pathways	[119]
In vitro inflammation models	EGCG treatment	↓ TNF-α, IL-6, IL-8, IL-1β via NF-κB inhibition	[120]
Keratinocytes subjected to ID-like stimuli	EGCG (100 µmol/L)	↓ elevated levels of CXCL10 and MxA	[121]
Wound healing/skin models	EGCGscaffolds/formulations	↓ IL-8, TNF-α; ↑ VEGF, angiogenesis, antioxidant enzymes	[122]
Nanoformulations with phosphatidylcholine/curcumin	EGCG combinations	↑ antioxidant and anti-inflammatory effects	[123, 124]
Cancer and chemotherapy models	EGCG treatment	↓ ROS-related DNA damage; ↓ NF-κB/STAT3 signaling; ↑ anticancer effects	[125]
Hepatic REDOX regulation models	EGCG nanoparticles	↑ antioxidant stability; ↑ Nrf2-mediated regulation	[126]
Cardiac hypertrophy models	EGCG (HDAC1 inhibition)	↑ TFAM, FUNDC1, mitochondrial DNA; ↑ mitophagy; improved cardiac function	[127]
SAH-induced mitochondrial damage	EGCG (50 µM)	Preserved membrane potential; balanced Drp1, OPA1, Mfn1/2, Fis1; ↑ autophagy; ↓ apoptosis	[128]
CA/CPR model	EGCG (9 mg/kg)	↓ ERK, cleaved caspase-3; ↑ ATPase, ATP content; ↓ ROS, mPTP opening	[129]
Alzheimer’s (AβPP/PS-1 mice)	EGCG treatment	Restored mitochondrial function (50–85%); normalized ROS, ATP, membrane potential	[130]
Osteoclast differentiation	EGCG (50 µM, 24–48 h)	↓ mitophagy markers; inhibited differentiation via AKT and p38MAPK	[131]
DOX-induced mitochondrial dysfunction	EGCG	↑ mitochondrial membrane potential; ↓ Drp1; metabolic shift to oxidative phosphorylation	[132]
Seizure models (SE-induced neuronal loss)	EGCG (50 µM)	Preserved DRP1-mediated fission; ↓ NF-κB Ser536 phosphorylation	[133]
Heat stress in porcine preadipocytes	EGCG (50 µM)	↓ fat accumulation via HSP70 targeting and AMPK-SIRT1-PGC-1α activation	[134]
Type 2 diabetic rats	EGCG (40, 80 mg/kg, 8 weeks)	↑ LC3, Beclin1, p-AMPK; ↓ p-mTOR; activated autophagy	[136]
Cellular models (autophagy)	EGCG treatment	↑ autophagosomes, autophagic flux	[136]
Stroke models	EGCG (20 µM for 12 h)	↓ autophagy via AKT/AMPK/mTOR modulation	[137]
rcccDNA mice (chronic infection)	EGCG (25 mg/kg, i.p., 42 days)	↓ HMGB1; promoted autophagic degradation	[138]
HAECs (lipotoxicity models)	EGCG derivative (E10)	↑ AMPK activation; improved autophagic flux; ↓ senescence and oxidative apoptosis	[139]
TGF-β1-treated cells (fibrosis models)	EGCG (60 µM for 4 h)	↑ autophagy (LC3-II/I ratio); ↓ α-SMA expression	[140]
LPS-induced pneumonia models	EGCG (15 mg/kg, i.p for 3 h)	↑ LC3, BECN1; ↓ mTOR; enhanced lung tissue autophagy	[141]
NR3C1-enhanced β cells	EGCG treatment	Promoted FTO degradation; ↓ oxidative stress; prevented excessive autophagy	[142]
HUVECs (vascular endothelial models)	EGCG (1, 5, 10 µmol/L for 24 h)	↑ Atg5, Atg7, LC3 II/I, Atg5–Atg12; ↓ PI3K-AKT-mTOR signaling and apoptosis	[143]
Caco-2 cells and fibroblasts	EGCG (10 µg/mL, 98 days)	↑ telomere-shortening-induced senescence and ↑ telomere-independent genotoxicity.	[144, 145]
H9c2 cells	EGCG (50 and 100 mg/L) for 24 h; then H₂O₂ (200 µmol/L) for 12–24 h	EGCG did not affect H₂O₂ levels but significantly inhibited H₂O₂-induced apoptosis (↓ Chromatin condensation, ↓ DNA fragmentation, ↓ apoptotic bodies, ↓ apoptotic rate); prevented telomere shortening, ↓ TRF2 loss, ↓ p53 and p21 upregulation.	[146]

## Thymoquinone

Thymoquinone (TQ) has been extensively studied for its pharmacological properties, including cardioprotective, anti-cancer, and neuroprotective effects. The increasing body of evidence suggests that TQ may hold considerable promise in modulating key cellular mechanisms involved in aging, such as oxidative stress, inflammation, autophagy, and mitochondrial dysfunction. TQ has been shown to inhibit the activity of various enzymes involved in the production of reactive oxygen species (ROS), thus reducing oxidative damage. Additionally, TQ activates cellular defense systems, including antioxidant enzymes, to enhance cellular resilience against oxidative stress [147]. In an in vivo study, TQ counteracted chlorpyrifos-induced disruptions in inflammatory and oxidative signaling, enhanced antioxidant defenses, including GSH, SOD, and CAT, and reduced the oxidative stress marker MDA. Furthermore, TQ suppressed the overproduction of inflammatory mediators [148]. TQ repressed pro-inflammatory cytokines such as TNF-α, IL-1β, IL-6, and iNOS. Conversely, it promoted anti-inflammatory cytokines like IL-10, demonstrating its role in rebalancing immune responses [149, 150]. Venkataraman et al. reported that TQ exerts its anti-inflammatory effects through activation of PPAR-γ, inhibition of MAP kinases, and suppression of NF-κB, as evident in both in vivo colonic inflammation and in vitro HT-29 cell models [150].

TQ further downregulated pro-inflammatory cytokines (IFN-γ, IL-17, IL-6) and upregulated anti-inflammatory cytokines (IL-4, IL-10, TGF-β), particularly influencing Th2 and Treg responses in an in vivo experimental autoimmune encephalomyelitis (EAE) model. Through real-time PCR, it was observed that TQ suppressed the expression of pro-inflammatory transcription factors such as T-bet and ROR-γt, and concurrently promoted the expression of Foxp3 and GATA3, which are markers associated with regulatory and anti-inflammatory immune responses [151]. TQ also enhances antioxidant defense mechanisms via Nrf2 activation and increased HO-1 expression, while inhibiting COX-2 and reducing oxidative and nitrosative stress [152]. Nano-formulations of TQ (e.g., chitosan nanocomposites, oleic acid-based vesicles) further amplify its anti-inflammatory efficacy by improving bioavailability and tissue targeting [153, 154].

### Autophagy

Thymoquinone has been reported to induce autophagy via the AMPK/mTOR/ULK1-dependent signaling pathway in C57BL/6 N mice with high-fat diet (HFD)-induced nonalcoholic fatty liver disease (NAFLD) [155]. Thymoquinone has been shown to protect cardiomyocytes from doxorubicin-induced apoptosis by enhancing autophagy through activation of the LKB1/AMPK signaling pathway [156]. Thymoquinone has been shown to enhance autophagy, thereby promoting the clearance of damaged cellular components and maintaining cellular homeostasis. In vitro and in vivo studies have demonstrated that TQ can activate autophagic pathways by inducing the expression of autophagy-related genes, such as Beclin-1, LC3-II, and ATG5. This activation is believed to be mediated through the inhibition of mTOR, a key negative regulator of autophagy [157]. In the PCO model, thymoquinone countered abnormal autophagy by downregulating key markers such as Beclin1, MAP-LC3-II, and the inflammatory mediator p65 [158]. Combined treatment with TQ and quercetin amplified autophagy-related responses by promoting Atg7 expression and upregulating the SIRT1/AMPK pathway more effectively than individual treatments [159]. TQ disrupts autophagic flux by blocking autophagosome-lysosome fusion, resulting in increased autophagosome presence and initiating apoptotic pathways [160]. Through PPAR-γ activation and interaction with 14-3-3γ, TQ counters the autophagic suppression in AngII-treated cardiomyocytes, restoring LC3 expression and lysosomal function [161] Pre-treatment of BEAS-2B cells with 20 and 50 µM TQ for 1 h TQ reduced the activation of LC3II, p-Drp, and necroptosis pathways, while maintaining the stability of PINK-1 expression [162].

### Mitochondria function

Administering 10 µmol/L TQ prior to CLZ exposure significantly mitigated oxidative and mitochondrial damage, as evident by stabilized ΔΨm, decreased ROS and MDA levels, restored SDH and GSH activity, and suppressed GSSG accumulation [163]. Prevention of arsenic-induced mitochondrial dysfunction was observed in Wistar rats pretreated with TQ (2.5 and 5 mg/kg b.wt.; p.o.), which effectively inhibited the decline in mitochondrial membrane potential (Δψm) [164]. TQ protects HaCaT skin cells from UVA-induced mitochondrial damage and apoptosis through NrF2/ARE pathway activation and COX-2 inhibition [165]. Cisplatin (CIS)-induced skeletal muscle atrophy is associated with mitochondrial dysfunction and increased levels of mitofusin-2 (Mfsn-2), tumor necrosis factor-alpha (TNF), and caspase-3 (Casp3), along with reduced meteorin-like (MtrnL) immunoreactivity, a marker linked to energy metabolism. Treatment with TQ (10 mg/kg/day via oral gavage) significantly downregulated the elevated Mfsn-2, TNF, and Casp3 expressions caused by CIS in rats, indicating its anti-inflammatory and anti-apoptotic effects on muscle tissue. Moreover, TQ restored the decreased MtrnL levels, suggesting a potential role in correcting energy metabolism and preserving mitochondrial function in CIS-induced muscle atrophy [166]. In 3-NP-treated rats, NanoTQ promoted mitochondrial recovery by enhancing PGC-1α-driven biogenesis and restoring mitochondrial complex enzyme activity. At the same time, it rebalanced neurotrophic and antioxidant signaling in the striatum by upregulating BDNF and GDNF, increasing Nrf2 and HO-1 expression, and downregulating Keap1 [167]. Following exposure to 6 Gy of radiation, mice pretreated with TQ exhibited reduced levels of IAP-1, IKBa, NFKB (p50), and iNOS in the spleen and brain, indicating attenuation of inflammatory and proliferative gene expression [168].

### Telomere maintenance

Thymoquinone can promote telomere shortening by suppressing telomerase enzyme activity in glioblastoma cells [169]. TQ (80 µM) also effectively reduced hTERT mRNA expression in CAL-62 and CGTH-W1 cells, with Genistein co-treatment enhancing this suppression to 6.5-fold and 3-fold reductions, respectively, after 72 h. Lower combination doses also decreased hTERT mRNA but lacked statistical significance [170]. Moreover, telomerase inhibition was confirmed in leukemia cells following treatment with TQ complexed with SBE-β-CD [171] (Table 4).

**Table 4:** A summary of thymoquinone’s effects on aging-related pathways

Model	Treatment/Duration	Main findings (↑ ↓ markers)	References
CPF-induced neurotoxicity in Wistar rats	thymoquinone (10 mg/kg)	↑ GSH, SOD, CAT; ↓ MDA, inflammatory mediators	[148]
Inflammation models	thymoquinone (20 and 40 mg/kg body weight for 24 h)	↓ TNF-α, IL-1β, IL-6, iNOS; ↑ IL-10	[149, 150]
Colonic inflammation and HT-29 cells	Thymoquinone (20 mg/kg/day)	↑ PPAR-γ activation; ↓ MAPK, NF-κB signaling	[150]
In animal model of Experimental Autoimmune Encephalomyelitis (EAE)	Thymoquinone (2, 10, and 20 mg/kg for 25 days)	↓ IFN-γ, IL-17, IL-6; ↑ IL-4, IL-10, TGF-β; ↑ Foxp3, GATA3	[151]
General oxidative stress models	thymoquinone	↑ Nrf2, HO-1; ↓ COX-2, oxidative stress	[152]
Nanoparticle systems	Nano-thymoquinone	↑ Bioavailability, anti-inflammatory efficacy	[153, 154]
high-fat diet (HFD)-induced nonalcoholic fatty liver disease (NAFLD) in C57BL/6 N mice	thymoquinone	↑ AMPK/ULK1; ↓ mTOR; promoted autophagy	[155]
doxorubicininduced H9c2 cell apoptosis	thymoquinone	↑ LKB1/AMPK; protected against DOX toxicity	[156]
In vitro/in vivo autophagy models	thymoquinone	↑ Beclin-1, LC3-II, ATG5; ↓ mTOR	[157]
PCOS model	Thymoquinone (TQ)	↑ Aromatase expression; ↓ Androgen Receptor (AR) levels; ↓ Autophagic markers; ↓ p65 levels; simulated super-ovulated condition.	[158]
Combination treatment	thymoquinonel + Quercetin(TQ 10 mg.kg − 1.d − 1, quercetin 50 mg.kg − 1.d − 1)	↑ Atg7, SIRT1/AMPK pathway	[159]
BEAS-2B cells	TQ (20–50 µM)	↓ LC3II, p-Drp, necroptosis; ↑ PINK-1 stability	[162]
Mitochondrial damage (CLZ model)	TQ (1, 5, and 10 µmol/l for 6 h)	↓ ROS, MDA; ↑ SDH, GSH; stabilized ΔΨm	[163]
Arsenic-induced mitochondrial dysfunction	Thymoquinone (2.5–5 mg/kg)	Preserved ΔΨm, mitochondrial protection	[164]
UVA-induced skin damage	TQ	↑ Nrf2/ARE pathway activation; ↓ COX-2	[165]
Cisplatin-induced muscle atrophy in rats	thymoquinone (10 mg/kg/day orally)	↓ Mfsn-2, TNF, Casp3; ↑ MtrnL, mitochondrial function	[166]
3-NP-induced neurotoxicity	NanoTQ (10 and 20 mg/kg) for 14 days	↑ Mitochondrial complex enzyme activity; ↑ PGC-1α (mitochondrial biogenesis); Restored BDNF and GDNF signaling; ↑ Nrf-2, ↑ HO-1, ↓ Keap1.	[167]
Radiation-induced injuryin mice	thymoquinonel pre-treatment (10 mg/kg for 24 h)	↓ IAP-1, IKBa, NF-κB (p50), iNOS in spleen and brain	[168]
Glioblastoma cells	thymoquinone	↓ Telomerase activity, telomere shortening	[169]
Thyroid carcinoma cells (CAL-62, CGTH-W1)	thymoquinone (80 µM) + Genistein	↓ hTERT mRNA (6.5-fold, 3-fold)	[170]
Leukemia cells	thymoquinone-SBE-β-CD complex	↓ Telomerase activity	[171]

## Conclusion

The growing body of evidence highlights the therapeutic promise of natural bioactive compounds such as curcumin, EGCG, thymoquinone, and resveratrol in mitigating the biological hallmarks of aging. These phytochemicals exert multifaceted actions by enhancing antioxidant defenses, suppressing chronic inflammation, improving mitochondrial function, and promoting cellular homeostasis. By modulating critical signaling pathways, they help attenuate cellular senescence, delay degenerative processes, and potentially extend healthspan. Their ability to engage in molecular cross-talk with age-associated regulatory networks positions them as compelling candidates for dietary geroprotective strategies. Continued translational research, including long-term clinical studies and optimized delivery systems, is essential to fully harness their longevity-enhancing potential and to pave the way for integrative approaches to healthy aging.

## Data Availability

No datasets were generated or analysed during the current study.

## Abbreviations

CLOCK: Circadian Locomotor Output Cycles Kaput
CRY: Cryptochrome
PER2: Period Circadian Regulator 2
CRY2: Cryptochrome Circadian Regulator 2
SA-β-gal: Senescence-Associated Beta-Galactosidase
PCNA: Proliferating Cell Nuclear Antigen
p16: Cyclin-Dependent Kinase Inhibitor 2 A
SIRT1: Sirtuin 1
NRF2: Nuclear Factor Erythroid 2–Related Factor 2
NRF1: Nuclear Respiratory Factor 1
sMaf: Small Maf Proteins (NRF2 co-factors)
CAT: Catalase
SOD: Superoxide Dismutase
SOD1: Superoxide Dismutase 1 (cytoplasmic)
SOD2: Superoxide Dismutase 2 (mitochondrial)
GSH: Glutathione
GPx: Glutathione Peroxidase
GR: Glutathione Reductase
ROS: Reactive Oxygen Species
MDA: Malondialdehyde
LDH: Lactate Dehydrogenase
LPO: Lipid Peroxidation
ER stress: Endoplasmic Reticulum Stress
LC3-II: Microtubule-Associated Protein 1 Light Chain 3-II
Beclin1 (BECN1): Autophagy Related Gene Beclin 1
3-MA: 3-Methyladenine (Autophagy Inhibitor)
p62: Sequestosome 1 (SQSTM1)
Atg5: Autophagy Related 5
Atg7: Autophagy Related 7
FUNDC1: FUN14 Domain-Containing Protein 1
TNF-α: Tumor Necrosis Factor Alpha
IL-6: Interleukin 6
IL-8: Interleukin 8
IL-1β: Interleukin 1 Beta
MCP-1: Monocyte Chemoattractant Protein-1
CXCL10: C-X-C Motif Chemokine Ligand 10
IFN-γ: Interferon Gamma
VCAM-1: Vascular Cell Adhesion Molecule 1
E-selectin: Endothelial-Leukocyte Adhesion Molecule 1
NF-κB (p65): Nuclear Factor Kappa-light-chain-enhancer of Activated B Cells (p65 subunit)
RELA: v-rel Avian Reticuloendotheliosis Viral Oncogene Homolog A (NF-κB subunit)
STAT1: Signal Transducer and Activator of Transcription 1
AKT: Protein Kinase B
p38MAPK: p38 Mitogen-Activated Protein Kinase
ERK1/2: Extracellular Signal–Regulated Kinases 1/2
JNK: c-Jun N-terminal Kinase
mTOR: Mammalian Target of Rapamycin
AMPK: AMP-activated Protein Kinase
p-AMPK: Phosphorylated AMPK
iNOS: Inducible Nitric Oxide Synthase
PDE4D: Phosphodiesterase 4D
PKA: Protein Kinase A
DYRK1A: Dual Specificity Tyrosine Phosphorylation Regulated Kinase 1 A
EGFR: Epidermal Growth Factor Receptor
TLR2: Toll-Like Receptor 2
MyD88: Myeloid Differentiation Primary Response 88
γH2AX: Phosphorylated H2A Histone Family Member X
p-BRCA1: Phosphorylated Breast Cancer Type 1 Susceptibility Protein
RAD51: RAD51 Recombinase
APE1/Ref-1: Apurinic/Apyrimidinic Endonuclease 1/Redox Factor-1
PPARα: Peroxisome Proliferator-Activated Receptor Alpha
GABPA: GA Binding Protein Transcription Factor Alpha Subunit
L1-ORF1: Long Interspersed Nuclear Element-1 Open Reading Frame 1
DJ-1: Parkinson Disease Protein 7
MEKK1: MAPK/ERK Kinase Kinase 1
TFAM: Mitochondrial Transcription Factor A
OPA1: Optic Atrophy 1
Mfn1/2: Mitofusin 1/2
Fis1: Mitochondrial Fission 1 Protein
mPTP: Mitochondrial Permeability Transition Pore
ATPase: Adenosine Triphosphatase
NO: Nitric Oxide
VO2max: Maximal Oxygen Uptake
HR: Heart Rate
NGFR: Nerve Growth Factor Receptor
FTO: Fat Mass and Obesity-Associated Protein
TRF2: Telomeric Repeat-Binding Factor 2
hTERT: Human Telomerase Reverse Transcriptase
LaminB: Lamin B
α-SMA: Alpha-Smooth Muscle Actin
SMAD3: Mothers Against Decapentaplegic Homolog 3
GAB1: GRB2-Associated Binding Protein 1
Ins2: Insulin 2
Pld2: Phospholipase D2
A20/TNFAIP3: Tumor Necrosis Factor Alpha-Induced Protein 3
LC3: Microtubule-Associated Protein 1 Light Chain 3
Beclin1 (BECN1): Autophagy Regulator
Atg5–Atg12: Autophagy-Related Proteins Complex
cleaved caspase-3: Activated Form of Caspas
